# Magnesium Transfer between Atomic Force Microscopy Probes and Metal Electrodes in Aqueous Alginate Electrolytes

**DOI:** 10.3390/polym16121615

**Published:** 2024-06-07

**Authors:** Walter J. Legerstee, Lindah Kiriinya, Mark Kwakernaak, Erik M. Kelder

**Affiliations:** 1 Department Storage of Electrochemical Energy, Reactor Institute Delft, Delft University of Technology, Mekelweg 15, 2629 JB Delft, The Netherlands; 2 Department of Electrical and Information Engineering, Faculty of Engineering, University of Nairobi, Harry Thuku Street, Nairobi 00100, Kenya

**Keywords:** magnesium deposition, magnesium alginate, atomic force microscopy, aqueous electrolyte

## Abstract

The upcoming energy transition requires not only renewable energy sources but also novel electricity storage systems such as batteries. Despite Li-ion batteries being the main storage systems, other batteries have been proposed to fulfil the requirements on safety, costs, and resource availability. Moving away from lithium, materials such as sodium, magnesium, zinc, and calcium are being considered. Water-based electrolytes are known for their improved safety, environmentally friendliness, and affordability. The key, however, is how to utilize the negative metal electrode, as using water-based electrolytes with these metals becomes an issue with respect to oxidation and/or dendrite formation. This work studied magnesium, where we aimed to determine if it can be electrochemically deposited in aqueous solutions with alginate-based additives to protect the magnesium. In order to do so, atomic force microscopy was used to research the morphological structure of magnesium deposition at the local scale by using a probe—the tip of a cantilever—as the active electrode, during charging and discharging. The second goal of using the AFM probe technology for magnesium deposition and stripping was an extension of our previous study in which we investigated, for lithium, whether it is possible to measure ion current and perform nonfaradaic impedance measurements at the local scale. The work presented here shows that this is possible in a relatively simple way because, with magnesium, no dendrite formation occurs, which hinders the stripping process.

## 1. Introduction

Due to the transition to green energy sources, the storage of electrical energy is becoming increasingly important. As a result, the development of lithium-ion batteries has accelerated in the last decade, which have found applications in various devices from mobile phones and laptops to electric bicycles and vehicles. State-of-the-art commercial Li-ion batteries are approaching their theoretical capacities, and, in order to bring these batteries to their practical maximum, solutions are being sought to implement the negative electrode of pure lithium, allowing an ultra-high specific anode capacity (3862 mA·g^−1^) [1]. Their development is especially complicated by the dendrite-forming property of lithium when it is electrochemically deposited, which, after years of research, still poses a major safety challenge [2]. Although this development will further increase energy density, the increasing demand for energy storage solutions is a direct result of the energy transition and will lead to a shortage of the limited raw materials needed to assemble lithium-based batteries in the near future. In addition, a number of disadvantages of lithium-based batteries, such as high cost and safety, give significant impetus to the development of new types of batteries. As a result, scientists are looking for alternatives to lithium as an energy carrier, with special attention to the possibilities of metals such as sodium, aluminum, and magnesium [3]. The latter two in particular are interesting candidates for use as pure metal electrodes because experiments have shown that neither of them has a strong tendency to develop dendrites during the deposition process [4,5]. Of these two lithium alternatives, magnesium has the lowest atomic mass (Mg 24.305, Al 26,982) and the lowest reduction potential after lithium (Li—3 V, Mg—2.37 V, Al—1.68 V). In addition, the volumetric capacity of magnesium is high compared to that of lithium (Mg 3833 mAhcm^−3^, Li 2046 mAhcm^−3^; in contrast, aluminum is superior, at 8046 mAhcm^−3^). These properties, together with the high abundance (magnesium is the eighth most abundant element) and the low cost, make secondary magnesium batteries attractive alternatives to lithium-ion batteries. However, in addition to the development of high-performance electrode materials, the challenges for Mg-based batteries mainly include finding a suitable electrolyte and overcoming the formation of passivation layers on magnesium electrodes. In contrast to the solid electrolyte interphase (SEI) that forms on the anode surfaces in lithium-ion batteries, a layer forms on metallic magnesium electrodes that completely blocks ions [6]. Preventing the formation of a passivation layer is therefore a crucial factor in the composition of electrolytes for magnesium batteries. The development of nonaqueous electrolytes is hindered by their poor cathode kinetics and complex chemistry [7]. The presence of a very small amount of H_2_O in these electrolytes can decompose the electrolyte; on the other hand, recent research showed that the presence of water can improve the kinetics at the cathode [8]. These conflicting findings make it interesting to look at aqueous electrolytes and to consider the presence of water in the electrochemical system of a magnesium battery. The alternative we studied is an aqueous electrolyte based on alginate, a natural hydrophilic polymer extracted from seaweed. We used this to electrochemically deposit Mg on different substrates. Atomic force microscopy (AFM) was then employed to research the reversible deposition of magnesium and investigate the morphological structure of the deposited magnesium at the local scale. As we worked with lithium in a previous study [9], we used the probe, the tip of the cantilever, as an active electrode. In the study presented here, the tip of the probe functioned as a point source, providing insight into the distribution of the deposited magnesium over an (infinitely) large surface area. We then used the results obtained under these highly controlled conditions at the macro scale and performed stripping and deposition experiments on different substrates.

### 1.1. Alginate Structure

**Figure 1:**
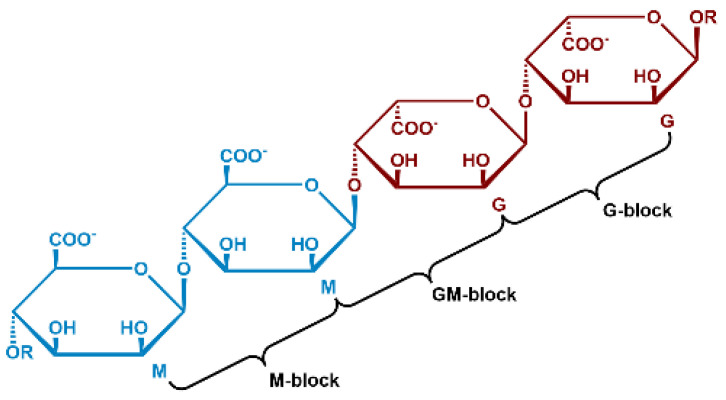
Structure of alginate polymers with monomer mannuronic (M) and guluronic (G) units.

Alginic acid is a polysaccharide that is abundantly available, environmentally friendly, cost-effective, and nontoxic. It is found in the cell walls of brown algae and is composed of two anionic monomers: (1,4) linked α–L guluronic acid (G) and (1,4) linked β–D–mannuronic acid (M) (Figure 1). The carboxyl group in the G monomer has the same orientation as the hydroxyl group, whereas in the M monomer the carboxyl group is oriented perpendicular to the hydroxyl group. Alginic acid is well known for its ability to bind multivalent cations, very efficiently forming alginate hydrogels in aqueous environments [10]. Upon deprotonation, the negatively charged carboxylate (COO^-^) can chelate with cations. Multivalent cations can ultimately crosslink the alginate polymer chains, increasing the viscosity of the solution and, in most cases, resulting in the formation of a hydrogel. Gel formation was shown to be greatly influenced by the interactions of the G blocks [11]. The linkage of two G monomers creates a cavity, making it an ideal place (cage) for a multivalent cation to reside. The crosslinking of the G blocks by multivalent cations creates a tightly held junction, popularly referred to as an ‘egg-box model’ [11].

The binding of the cations to alginate is influenced by the cation’s properties: its charge, affinity to water, ionic radius, and chemical affinity with the alginate. Bivalent alkaline earth cations (Mg^2+^, Ca^2+^, and Sr^2+^) typically form ionic bonds. In contrast, bivalent transition metal ions (Mn^2+^, Co^2+^, Cu^2+^, Fe^2+^, and Zn^2+^) and trivalent metal cations (Fe^3+^, Cr^3+^, Al^3+^, Ga^3+^, Sc^3+^, and La^3+^) usually form complex uronates via strong coordination–covalent bonds [12]. Alginate’s affinity for these bivalent cations was shown to increase as follows: Pb > Cu > Cd > Ba > Sr > Ca > Co, Ni, Zn, Mn > Mg [11]. Mg-alginate has long been considered as a nongelling alginate; however, studies found that Mg induces gelation in high-G-content polymers with longer gelation times [13]. Mg-alginates gels are, however, not stable in water and dissolve quickly. The present work investigated the transfer of magnesium between the AFM probes and magnesium metal electrodes in an aqueous Mg-alginate electrolyte.

### 1.2. Magnesium Surface Reactions

In general, magnesium dissolves in aqueous environments via the following anodic reaction,

(1) $${Mg}_{\left(s\right)}\to {Mg}_{\left(aq\right)}^{2+}+2e^{-}$$

which is followed by

(2) $$2H_{2}O_{\left(l\right)}+2e^{-}\to H_{2\;(g)}+2{\left(OH\right)}_{\left(aq\right)}^{-}$$

Due to the rise in the pH on magnesium’s surface, the following reaction forms magnesium hydroxide, which can precipitate on magnesium’s surface [14]:

(3) $${Mg}_{\left(aq\right)}^{2+}+2{\left(OH\right)}_{\left(aq\right)}^{-}\to Mg(OH{)}_{2\;(s)}$$

The result is perceived as a dark layer covering the surface of the magnesium [14,15]. 

## 2. Experimental Details

### 2.1. Mg-Alginate Electrolyte Synthesis 

Magnesium alginate was synthesized using alginic acid (Alg-acid; VWR International, Amsterdam, The Netherlands) and magnesium hydroxide (Mg(OH)_2_; (Merck Sigma, Amsterdam, The Nethelands). Alg acid powder was first weighed, and 20 mL of methanol was added to one equivalent quantity of the Alg acid. The addition of methanol served to dissolve the Alg acid. To deprotonate the Alg acid, ammonia was added, producing a thick slurry. In a separate beaker, Mg(OH)_2_ was weighed, and 5 mL of methanol was added to ean quivalent quantity of Mg(OH)_2_. The mixture was then put in a sonication bath to accelerate the dissolution. The powder was then added to the Alg acid slurry and stirred for one day at a constant temperature of 40 °C in order to minimize alginate decomposition. After decanting, the residue was air-dried for 4 days and then vacuum-dried for another 2 days. The vacuum-drying process yielded a solid Mg-alginate. The solid Mg-alginate was ball-milled to a powder in a planetary ball mill (Fritsch, Pulverisette 7, Ede, The Netherlands). The Mg-alginate was crushed in the ball mill for 15 h at 240 RPM. The resulting Mg-alginate powder (Mg-Alg-P) was used to prepare the electrolyte. In order to do so, 2 w/o Mg-Alg-P was dissolved in water, together with 5 w/o NaCl and 5 w/o MgCl_2_·6H_2_O, where the Cl^-^ ions acted as the conducting electrolyte ions for charge compensation. 

### 2.2. Sample Preparation for Deposition and Stripping Experiments

Deposition experiments were performed on flat substrates to avoid the preferred growth of magnesium on the seed-like features on the surface of the sample. As a nonmagnesium substrate, we used single-sided highly polished aluminum (thickness 0.8 mm, Ra < 0.05 µm). An accurate and flat sample of pure magnesium was obtained by means of sputter deposition. A silicon wafer (Sigma-Aldrich, St. Louis, MO, USA; <100>, 50.8 mm diameter, 0.5 mm thickness, essentially without dopants) was cleaned with acetone and isopropanol and placed into a sputter deposition system (AJA Int. ATC 1800, Hingham, MA, USA) with a pressure of <10^−8^ mBar and a high-purity Mg target (Kurt J. Lesker, East Sussex, UK), 99.95% pure, 2” diameter × 0.25” thick). After plasma cleaning (5 min in argon at 20 mBar and an RF power of 24 W),a layer of magnesium of 500 nm was applied (26 min in argon at 5 mBar and an RF power of 100 W). The silicon wafer sputtered with magnesium was then transferred to a glove box in an airtight container and stored there until needed for the experiments.

### 2.3. Electro-Chemical Experiments Using AFM

In our previous work [9], we described how we used AFM to perform local electrochemical measurements on the electrodes of lithium-ion batteries at the submicron to nano scales. We developed several methods to provide the tip of the AFM probe with a small amount of lithium so that nonfaradaic measurements can be performed. In the work presented here, we used one of these techniques, the thin-film method, to investigate reversible magnesium deposition between the probe tip and substrate with the use of the prepared alginate-based electrolyte. 

We used a similar AFM system (NT-NDT NTegra P9, Moskow, Russia) as in our previous study, with the main difference being that this setup was not housed in a protected glovebox environment due to conducting experiments with aqueous solutions. The used AFM had an accessible design for sample handling and was coupled with a galvanostat/potentiostat (Metrohm Autolab PG-STAT302F, Utrecht, The Netherlands) to perform the electrochemical experiments between the tip and substrate. All deposition experiments with the AFM probe as an electrode described here were performed with a diamond-coated conductive probe (Nanosensors GmbH CDT-NCHR, Neuchatel, Switzerland) with a nominal resonance frequency of around 400 kHz and a nominal force constant of around 80 N·m^−1^. 

For detecting the presence of magnesium on the probe tip, we used the method described in our previous work [9]. Similarly, in the work presented here, the addition or removal of magnesium to or from the tip was monitored by measuring the resonance frequency of the cantilever after the experiment. For this purpose, the probe was lifted from the Mg-alginate film, and the AFM instrument was set in tapping mode. The resonance frequency could be determined automatically by a frequency sweep executed by the oscillating system. Since our goal was a qualitative analysis of the deposition of magnesium, we did not determine the amount of Mg (as we performed in our previous work) but used the resonance shift as an indication to determine whether deposition or tripping had occurred. 

**Figure 2:**
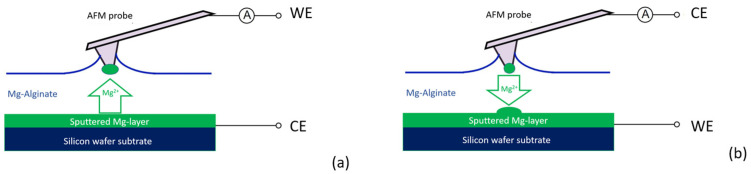
Active probe AFM experiments: (**a**) stripping the substrate and deposit on probe tip and (**b**) stripping the probe tip to deposit on substrate.

The magnesium transfer experiments were performed as shown schematically in Figure 2. For deposition to the probe tip, this probe tip was connected as the working electrode and the magnesium substrate as the counter electrode (Figure 2a) and vice versa for stripping magnesium from the probe tip to the substrate (Figure 2b). Mg-alginate was applied to the substrate by moving a piece of tissue material soaked with Mg-alginate over the surface, leaving a thin film on the substrate. The remaining film layer had a thickness of between 5 and 8 µm and completely covered the substrate surface. With the AFM setup operating in contact mode, the probe was moved slowly to the surface of the Mg-alginate film while accurately monitoring the moment of bending of the probe with the detector of the laser reflection signal. At the moment a small deviation of the probe was detected, the approach was immediately stopped, and the distance between sample and probe (*z* axis) was manually adjusted to improve the contact between the probe tip and electrolyte. When contact between the probe tip and electrolyte stabilized, a bias voltage was applied between tip and substrate to start the deposition process. The deposition was stopped by removing the bias voltage or lifting the tip of the probe from the electrolyte surface. 

For the reversable deposition experiment, we started with a fresh AFM probe and a flat and unused magnesium-sputtered wafer substrate carefully covered with Mg-alginate. After a short deposition time, the presence of a small amount of magnesium on the probe tip was confirmed by the shift in the resonance frequency of the cantilever. The substrate was then rinsed with demi water to remove the Mg-alginate solution, and the surface was investigated using AFM in imaging mode. A new substrate was mounted and carefully covered with Mg-alginate for the stripping experiment. Stripping of the tip was achieved by simply reversing the polarity between the tip and substrate (Figure 2b). Again, a shift in the resonance frequency of the cantilever was used to determine whether magnesium had been removed from the tip. After detecting a significant shift in the resonance frequency, the substrate was carefully rinsed clean with deionized water and then examined via scanning electron microscopy (SEM).

The deposition results on the substrates were examined using a different probe than used for electrochemical experiments: a more accurate nonconductive probe (Nano sensors PPP-NCHR, Neuchatel, Switzerland, resonance frequency 330 kHz, force constant 42 N/m, tip radius < 10 nm). Scanning electron microscopy (SEM; JEOL JSM-IT100, Zaventem, Belgium) and energy-dispersive spectroscopy (EDS) were used for investigating the results of deposition on the probe tip.

### 2.4. Bulk Stripping and Deposition Measurements

**Figure 3:**
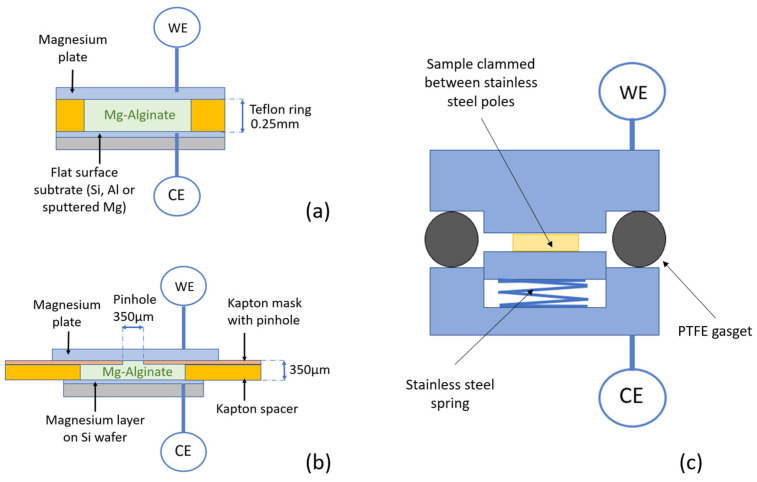
Bulk deposition experiments using Mg-alginate as an electrolyte. (**a**) Mg-alginate is situated between a magnesium plate as working electrode and a flat substrate as the counter electrode where deposition takes place. (**b**) Symmetrical cell with a mask on the stripping side. (**c**) Airtight laboratory cell consisting of airtight-mounted stainless-steel poles separated by a PTFE gasket where the sample is clamped between poles with a stainless-steel spring.

Figure 3 provides a schematic representation of the bulk experiments performed in a dismountable laboratory cell (Figure 3c) with high-purity magnesium foil (Sigma Aldrich; purity 99.99%, thickness 0.015 mm) as the counter and reference electrode and the deposition substrate as the working electrode. The electrodes were separated by a porous glass fiber membrane (thickness 0.25 mm) in the first experiments. Because the glass fiber appeared to have an influence on the deposition (see results below), the separator material was removed in follow-up experiments, and the spacing was obtained by applying a Teflon ring (thickness 0.25 mm, inner diameter 6 mm) between the layers, as shown in Figure 3a. 

To investigate the shape and distribution of the deposition more closely, a symmetrical cell (Mg plate on one side, sputtered Mg substrate on the other side) was provided with an extra mask, as shown in Figure 3b. On the working electrode side, an insulating mask (Kapton foil, thickness 50 µm) was provided with a pinhole (d = 350 µm) in the middle and stacked together with a separation ring (Kapton spacer, thickness 300 µm) and the deposition substrate (counter electrode).

For stripping/deposition experiments, the laboratory cell was connected to a Maccor^TM^ battery tester (Maccor 4000), which was used under constant-current conditions (galvanostatic). Deposition on the substrate side was carried out with a current density of 100 µA.cm^−2^ for 15 min, with a fixed potential limit of 0.2 V vs. Mg for all samples. After deposition, the laboratory cells were carefully dismounted and samples were rinsed with demi- water to remove residual alginate on the surface, which was followed by drying under a vacuum for 30 min. After this cleaning procedure, the result of deposition on the sample surface was investigated via SEM. For the non-Mg substrates, the presence of magnesium was confirmed with EDS.

## 3. Results and Discussion

### 3.1. Characterization of Mg-Alginate Solutions

**Figure 4:**
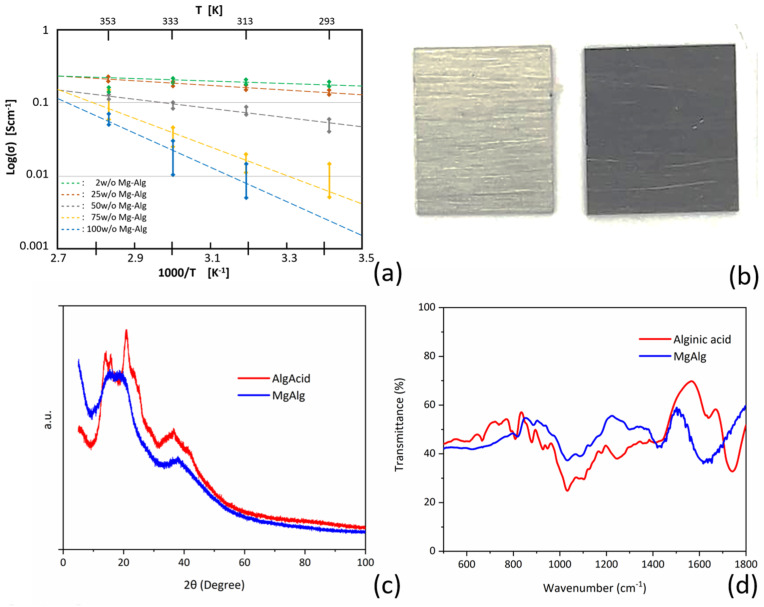
(**a**) Conductivity of electrolytes at different concentrations; (**b**) bare magnesium surface compared with surface covered by black layer after contact with alginate solution; (**c**) X-ray diffraction analysis of alginate acid (red) and magnesium alginate (blue); (**d**) Fourier transform infrared spectroscopy analysis of alginate acid (red) and magnesium alginate (blue).

The various Mg alginates and Mg-alginate aqueous solutions were characterized by X-ray diffraction (XRD: X’Pert Pro PANalytical, Malvern, UK) and Fourier transform infrared (FTIR: Thermo Scientific Nicolet iS50 FTIR, Bleiswijk, The Netherlands) spectroscopy, which showed that the solid samples did not contain any residual Mg(OH)_2_ or alginic acid (Figure 4c,d). 

### 3.2. Electrochemical Characterization

The ionic conductivity of the alginate samples was measured at different temperatures, as shown in Figure 4a. The conductivity for all concentrations of electrolyte generally increased with temperature. The Mg-alginate concentration influenced the conductivity strongly, with lower concentrations (2 wt.% and 25 wt.%) exhibiting higher conductivities, which can be understood by the strong dependency of Mg-ion mobility on concentration. Hence, the electrolyte conductivity seemed to rely more on the ionic mobility than the concentration of the Mg ions, e.g., addition of more charge carriers (Mg-alginate) did not improve the conductivity of the electrolyte.

**Figure 5:**
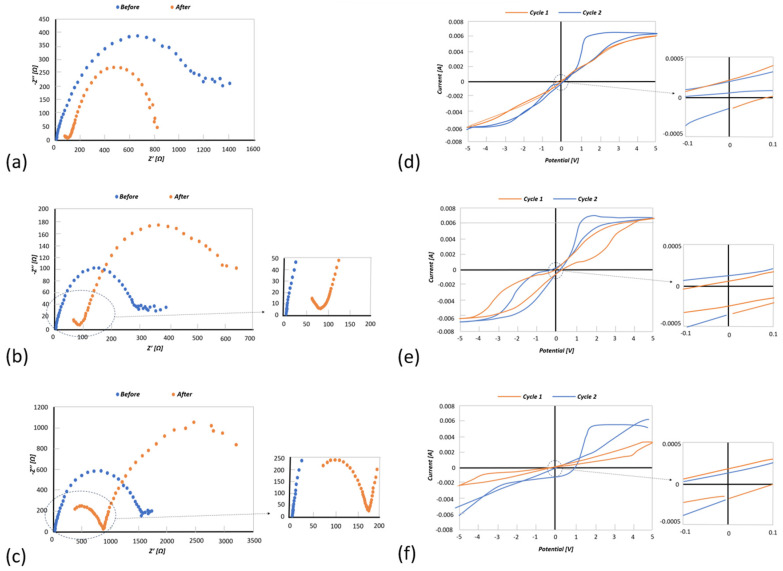
(**a**) EIS results for the 2 wt.% Mg-alginate electrolyte before (blue) and after (red) forming of the black layer; (**b**) EIS results for the 25 wt.% Mg-alginate electrolyte before (blue) and after (red) forming of the black layer; (**c**) EIS results for the 50 wt.% Mg-alginate electrolyte before (blue) and after (red) forming of the black layer; (**d**) CV curve for 2 wt.% Mg-alginate electrolyte before (blue) and after (red) forming of the black layer; (**e**) CV curve for 25 wt.% Mg-alginate electrolyte before (blue) and after (red) forming of the black layer and; (**f**) CV curve for 50 wt.% Mg-alginate electrolyte before (blue) and after (red) forming of the black layer.

The Mg-alginate concentrations with the most promising conductivity (2 wt.%, 25 wt.%, and 50 wt.%) were then chosen to perform cyclic voltammetry (CV) and electrochemical impedance spectroscopy (EIS) measurements (Metrohm Autolab PGSTAT12). For characterization, EIS analyses were used to measure the internal resistance of the electrolyte and the interfacial resistance between the electrolyte and the magnesium electrodes. While running EIS and CV, a dark layer was observed on the pristine magnesium electrodes (Figure 4b). This layer became more apparent with each experiment, so the effect of the layer on the system was investigated. The EIS profiles using 2 w/o (Figure 5a), 25 w/o (Figure 5b), and 50 w/o (Figure 5c) show a significant change in the overall impedance. This seemed to reduce the charge transfer resistance, causing a subsequent reduction in the impedance but introduced a new process with its own time constant, which was clearly seen from the additional semicircle formed in the high-frequency range (Figure 5a–c).

In Figure 5c–e, the CV curves for 2 w/o, 25 w/o, and 50 w/o are presented, respectively. Before formation of the black layer, the CV showed a low cycling efficiency due to the high impedance. After the formation of the black layer, the impedance reduced, causing the cycling efficiency to improve. However, it looked like it was limited by the number of charge carriers. The same behavior can be seen in Figure 5d, where cycling efficiency improves after the formation of the black layer. The CV curve for the 25 w/o electrolyte, however, did not seem to be affected by electronic conduction since the electrolyte contained more charge carriers. Figure 5f (black curve, before formation of black layer) presents a very low cycling efficiency due to the high impedance (see Figure 5c). The formation of the black layer did not seem to significantly improve the efficiency due to the increased interfacial and charge transfer resistances (see Figure 5c). 

### 3.3. Electrochemical Deposition of Magnesium Using AFM Probe as an Electrode

**Figure 6:**
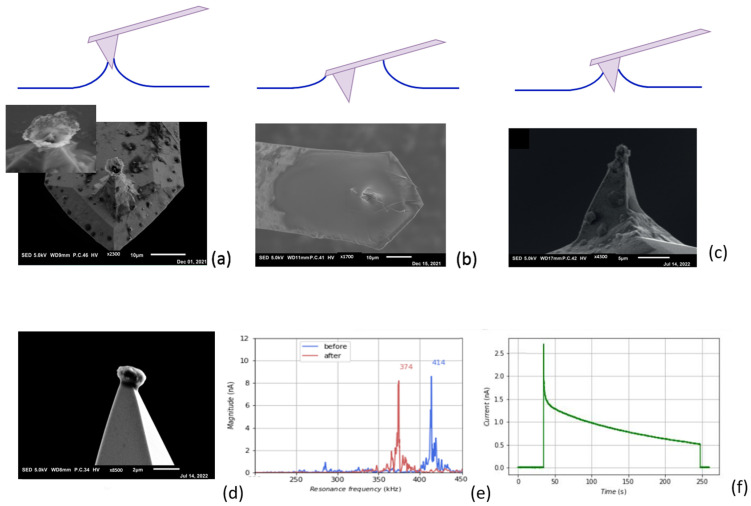
Fine tuning of the AFM settings for optimal deposition of Mg on the very end of the probe tip. (**a**) Very small electrolyte contact area results in cone-formed Mg deposition. Inset: Cone in detail (up) and a schematical impression on how the Mg cone is growing due to exhausting of Mg^2+^ ions; (**b**) electrolyte contact area covers part of the cantilever, which results in overall coverage of Mg on the cantilever; (**c**) correct settings resulting in a deposition on the tip-end; (**d**) subsequent deposition with the same settings as in (**c**); (**e**) shift in the resonance frequency of the cantilever caused by deposition of the tip; (**f**) registration of the current during deposition.

Magnesium deposition on the probe tip was performed as shown in Figure 2a. It should be noted that the performance of this process strongly depends on both the ion current and the contact area with the electrolyte. The latter is practically indeterminable in advance because the interaction between the probe tip and the electrolyte surface is influenced by a number of interdependent or variable parameters, such as the capillary forces between tip and liquid, the viscosity of the electrolyte, the deflection of the cantilever, and the setpoint of the control loop of the AFM. Therefore, the correct settings were found via trial and error until the right conditions occurred and magnesium was deposited in the desired manner on the probe tip. Figure 6a,b indicate two extremes of deposition on the probe tip. Figure 6a shows the situation when the probe landed on the electrolyte with a very sensitive adjustment so that the tip just touched the electrolyte and was not adjusted further. Only a small part of the liquid was in contact with the tip, resulting in a deposition of magnesium as a cone-shaped structure. This formation can be understood by the wetting of the tip, the deposition rate of magnesium, and the exhausting of Mg^2+^ ions in the solution in the formed cone at the tip, as depicted in the schematic in Figure 6a. It further proves that the deposition must be metallic magnesium; otherwise, electrons would not find their way to the rim of the cone. Figure 6b shows the situation when the probe had sunk just too deep into the electrolyte, with part of the cantilever in contact with the liquid. The deposition in the SEM photo shows complete coverage of the cantilever and the tip. Figure 6c shows the desired situation, where the tip just breaks through the liquid surface and deposition only takes place at the end of the probe tip. Once this situation is achieved, subsequent depositions can be reproduced with similar settings.

The AFM settings used for the deposition in Figure 6c appeared to give the best results and were also reproducible, as can be seen in one of the subsequent depositions with these settings (Figure 6d). Figure 6e shows a graph of the resonance frequency of the cantilever before and after deposition, and Figure 6f shows the measurement of the current between the tip and substrate. Both indicate the successful deposition during the experiment.

**Figure 7:**
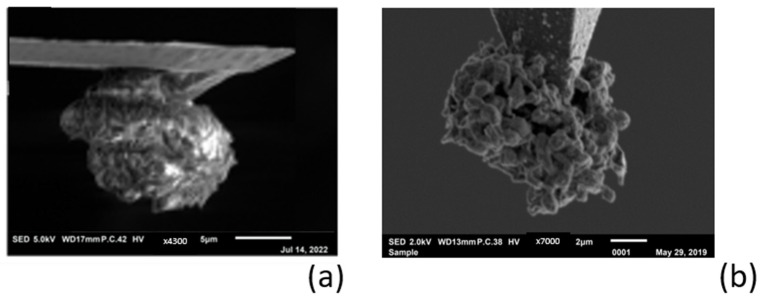
(**a**) Large amount of magnesium deposited on the tip; (**b**) lithium deposition on the tip [9].

AFM offers good opportunities to take a closer look at the morphological structure of a deposit, because the tip of the probe can be regarded as a point source. Deposition on a tip results in a freely positioned spherical shape that can be viewed all around with SEM. To obtain an impression of the morphological structure of the deposited magnesium when using the Mg-alginate electrolyte, a long-term magnesium deposition was performed, as can be seen in Figure 7a. For comparison, we show a similar deposition with lithium in Figure 7b (taken from our previous study, [9]). 

The difference in deposition morphologies between lithium and magnesium can be clearly seen in the side-view SEM pictures of the probe tip: Figure 7a shows a massive spherical magnesium deposit with a compact dendrite-free structure, while Figure 7b shows a lithium deposit with a porous and dendrite-shaped spherical structure. These experimental findings are theoretically supported by the modeling and DFT calculations in the works of Jäckle and co-authors [16,17], which describe how magnesium tends to grow with a smooth surface because it exhibits lower diffusion barriers than lithium. The dendrite formation that occurs with the electrodeposition of lithium is also influenced by the electrode/electrolyte interface upon long-term cycling. However, since the origin of dendrite formation is based on the characteristic differences between Li and Mg with regard to the elemental properties relevant for growth, it can be concluded that the properties of the electrolyte in the case of magnesium do not significantly influence dendrite growth. 

### 3.4. Reversable Magnesium Deposition between Probe Tip and Substrate

**Figure 8:**
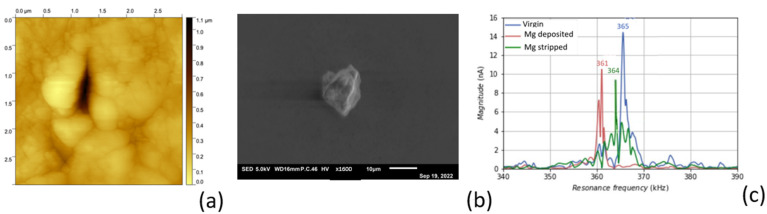
Deposition and stripping experiment: (**a**) AFM image; (**b**) SEM picture; (**c**) shift in resonance frequency.

In our previous study [9], we observed that it is not possible to electrochemically remove (strip) lithium from a shape in a dendrite structure (as shown in Figure 7b). Inspection with SEM showed that the magnesium deposited on the tip had a dense structure without any dendrite formation, which allowed for reversible magnesium deposition. First, magnesium was taken from the substrate and deposited on the probe tip, as schematically shown in Figure 2a. After this experiment, the surface of the substate was scanned with AFM using the accurate probe that was selected for surface investigation. The image in Figure 8a shows the spot where the magnesium was taken during the experiment. Obviously, as can be seen in this picture, magnesium was taken from a very small area, the size of which was comparable to the size of the probe tip. The probe with magnesium deposited on the tip was used for further experiments. Stripping of magnesium from the probe tip was performed as schematically shown in Figure 2b. Figure 8b shows an image of the magnesium feature that was electrochemically removed from the probe tip and deposited on the substrate. Again, the size and shape of the deposited material were the same as those of the probe tip, meaning that the ion current followed a very straight path through the electrolyte. Figure 8c gives the shift in the resonance frequency of the probe cantilever measured in tapping mode after each experiment. Although the distortion of the peak made it difficult to determine the precise shift, the clearly discernible change in resonance frequency gave a good indication of the addition and removal of mass at the tip. This experiment clearly shows that the reversible deposition of magnesium with an aqueous Mg-alginate-based electrolyte is possible.

### 3.5. Electro-Chemical Magnesium Deposition at Bulk Level

**Figure 9:**
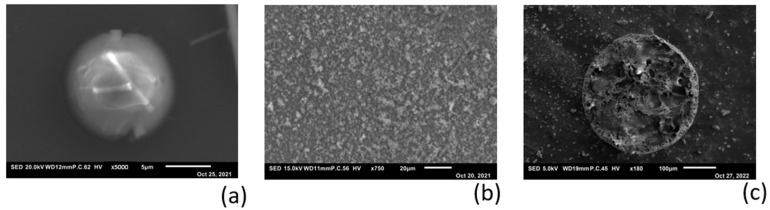
Deposition of Mg on different substrates with use of aqueous Mg-alginate electrolyte: (**a**) SEM picture of Mg on polished aluminum substrate. Inside the spherical particle, a small piece of glass fiber from the separator is visible, which acted as a seed for deposition. The inset image shows the EDX scanning result for Mg; (**b**) SEM picture of Mg on silicon wafer. Deposition without a separator shows random-oriented deposition on the substrate; (**c**) Mg deposited on sputtered Mg substrate using a mask with a pinhole of 350 µm.

The results of the AFM experiments on a submicron scale gave us a reason to perform magnesium deposition at the macro scale, as described in Figure 2. Figure 9 shows SEM pictures of the deposition of Mg on substrates of aluminum (Figure 9a) and silicon (Figure 9b). The experiment with aluminum (Figure 9a) was performed using a glass fiber separator between the substrate and stripping target. Due to the relatively high energy of the SEM electron beam used (20 keV), the seed on which the magnesium was deposited was visible inside the particle. To avoid the preferred deposition on a seed-like feature, further experiments were performed using the cell configuration in Figure 2a, where the separator material was replaced with a Teflon spacer. Figure 2b shows the apparently random deposition on a silicon substrate, where magnesium appears to have a slight preference for clumping or accumulating on previously deposited material.

To verify the sub-micron-scale findings in Figure 8b, where the ion current through the electrolyte appears to follow a very straight path, the macro-scale deposition on a flat magnesium substrate was performed using a mask (as schematically shown in Figure 2b). The SEM image in Figure 9c shows the result of this experiment, where the magnesium forms a cylindrical projection of the mask with the exact size of the pinhole (350 µm). The amount of magnesium that ended up next to the circular deposition was negligibly small. 

## 4. General Conclusions

In the study described here, we focused on the basic and critical phenomena that are needed to develop an alginate-based aqueous secondary magnesium battery. The synthesized alginate powder was characterized by XRD and FTIR (Figure 4c,d), after which different concentrations of Mg-alginate were dissolved in water and analyzed via EIS and CV. In general, we concluded that the aqueous Mg-alginate solution shows electrolyte activity. The 2 wt% Mg-alginate in water with an ion conductivity of ~1 mS.cm^−1^ was used further for testing.

A black layer formed on the interface between the aqueous Mg-alginate and the magnesium metal anode surface. The results indicated that this layer had a different composition than the MgOH_2_ normally formed at the interphase of magnesium and water because it did not seem to block Mg ions to and from the metal surface. However, the specific composition of the black layer was not fully understood because it was an amorphous layer. The layer may have had a composition consisting of magnesium, oxygen, carbon, and hydrogen. Further research using specific measuring techniques is required to characterize the precise composition of the layer. The black layer did not appear to have any adverse effect and may have acted as a passivation layer for the magnesium surface. Further research into this ion-permeable passivation layer and the contribution and necessity of the alginate in its formation is recommended.

We successfully created an active AFM probe by providing the probe tip with metallic magnesium. With this active probe, it is possible to apply controlled magnesium to a substrate by means of electrochemical deposition. The electrochemical deposition of magnesium using a Mg-alginate electrolyte was demonstrated. Because the AFM probe can be considered an electrode point source with respect to the infinite 2D and plate-shaped electrode substrate, it was possible to analyze the deposition of magnesium both on the tip and on the substrate. These measurements provided insight into the way in which magnesium ions were transported through the alginate electrolyte. It appeared that the magnesium had a strong preference for taking the shortest path, as evidenced by the shapes on both from stripping and deposition on the substrate (Figure 8a,b). The magnesium formed solid structures, unlike lithium, where unwanted dendrite formation occurs.

The strong preference for a nondendritic morphology of magnesium provides good opportunities to use this deposition/stripping method for measuring ion current, which we achieved in this work with a qualitative current signal (Figure 6f). It also opens the way for future researchers to perform nonfaradaic impedance measurements at a local scale. Unlike with the use of lithium, as we investigated in our earlier work [9], a small amount magnesium can be deposited on an AFM probe with a relatively simple electrodeposition process. This makes it technically less complicated to measure the ion current while scanning over a surface with a magnesium-containing probe tip and thus to create an active-probe AFM image. The development of this 2D ion current scanning probe method creates a new and powerful analysis technique in the palette of AFM -based measuring techniques. With advanced AFM equipment, cathode materials can be investigated in this way in shading mode, providing insight into the relationship between the ion current and morphology of the electrode material.

## Data Availability

Data are contained within the article.
